# Shame and Medicine: An Introduction

**DOI:** 10.1353/lm.2025.a975542

**Published:** 2025-01-01

**Authors:** Arthur Rose, Luna Dolezal

“Shame,” writes Elspeth Probyn, “is a painful thing to write about. It gets into your body. It gets to you.”^1^ The biggest worries, she continues, are that you might not get your point across, may fail to do your topic justice, could be called a sham—in other words, that your own interest in a topic exceeds your capacity to write about it. “Simply put, it is the challenge of making the writing equal to the subject being written about.”^2^ How much more so, when the subject of the writing is shame itself?

There is now considerable evidence that shame, a painful feeling or experience of inadequacy, lessness, or negative judgment, plays a significant role in medical practice, from clinical encounters through medical education to the social ecologies of hospitals, surgeries, and other places of healthcare delivery. Shame, “a feeling of being exposed to a gaze which produces a view of yourself which you cannot control,” in Imogen Tyler’s formulation, seems an inevitable consequence of what Michel Foucault famously called “the medical gaze,” that epistemic maneuver that subtracts the individual from their illness, paradoxically, by making them synonymous with their illness.^3^ Patients are, as Foucault noted, likely to be subjected to this control-depriving exposure. But the formation and disciplining processes that induct new healthcare professionals in the use of the medical gaze, and govern its continued use in their professional lives, also work through the constant threat of exposure and shame. This may be why, previously, “the subject of shame and humiliation in the medical care of patients [was] rarely discussed, studied, or written about,” its effacement “the elephant in the room.”^4^ Recently, however, there have been welcome signs of a shift, or, perhaps, a thaw, in its treatment as an object of study. Shame has been linked to chronic illness, clinical encounters, medical education, medical error, mental health, and professional identity formation.^5^ Proposals to identify shame as an affective determinant of health have turned into manifestoes for shame-sensitive practice and training programs for shame competence in healthcare.^6^

It is fitting that this burgeoning interest in shame and medicine—led, as it is, by collaborations between medical professionals and scholars in the medical and health humanities—should also prompt the responses from *Literature and Medicine* in this theme issue. After all, shame finds some of its most powerful treatments in literature, literary writing, and literary criticism. “Shame,” Timothy Bewes maintains, “is an event of writing”; there is, according to Barry Sheils and Julie Walsh, an “intrinsic relation between shame and writing”; shame, reflects Kaye Mitchell, “attends—inhabits, is associated with, is provoked by and sometimes inhibits—writing itself.”^7^ Or, to return to Sheils and Walsh, “the very act of writing—be it the private diary entry, the functional to-do list, or the crafted and much redrafted excerpt of literary prose—will inevitably leave on the page a residue or trace of shame.”^8^ This residue emerges, for Sheils and Walsh, in “an economy of affective transfer between writer, reader and text, operating in excess of representation,” wherein anxieties about what one hasn’t managed to write merge with those about what one has written, badly.^9^ For Bewes, it is a more fundamental incommensurability between the attempt to give form to shame and the inevitable inadequacy of such representational strategies. This means that, in addition to thematic or episodic *scenes of shame* in literature, it is important to address what Mitchell calls “the *formal* challenges and disruptions of presenting shame in literature” and attend to “the functions and effects of shame within and beyond particular texts.”^10^

Shame may be difficult to write about, but thinking of writers shouldering this difficulty, carrying it in their body, perhaps crystallizes the work that they do. As editors, we are thankful for the work the contributors have done for this theme issue—a gratitude we see reflected in their own attitudes to the writers they discuss. In thinking how the essays might best speak together, we thought at first to divide these essays into those that considered doctors (Dowland, Lusk), patients (Cheston, Hommes, Hustis), and relations between the two (Green), with further sections on people and conditions that have been medically pathologized (Abbott, Cooper, Heney) or medically constrained (Robison). We eventually decided against this course, because it seemed to reinstate divisions between doctors, patients, and those who live with stigma. Other pairings may have turned around medically unexplained symptoms (Cheston, Hommes), disability (Cooper, Hommes), or memoir (all, apart from Dowland, Cooper and Abbott). Was there another way, we wondered?

Instead, we have thought of the essays as presenting three overlapping responses to shame in its relation to *moments of becoming, acts of confession*, and *styles of mediation*. In our first cluster of essays, Harriet Cooper, Penelope Lusk, and Maaike Hommes consider the role shame plays in formative processes, as people become subjects and, particularly, are subjected to specific identities: the disabled person, the doctor, and the patient. In the first two essays of our second grouping, on confession, Harriet Hustis and Douglas Dowland attend specifically to the significance of the face for shame, and how face, as a social value, may be lost or saved, for the patient whose actual face is affected and for the doctor who operates on them. In the next two essays in that group, Katharine Cheston and Chloe Green consider the methods we use for researching shame: how we receive the intimate and vulnerable reflections of others, how we read them respectfully and with care. Across these essays, there’s a strong sense of the importance of the encounter, between doctor and patient, diagnoser and diagnosed. In the final group of essays, on mediations, Carla Robison, Traci B. Abbott, and Veronica Heney consider how specific processes, identities, and actions are medicalized, pathologized, and then stigmatized in cultural works about abortion from 1960s and 1970s, about trans life from the 1970s to the 2010s, and about self-harm from the 2010s to 2020s. Shame may not be the inevitable consequence of stigma—the marking of bodies as *other*—but, as they and others in the theme issue show, stigma creates the conditions of exclusion, inequality, and othering in which feelings of shame thrive.

We might circle back, then, to the rise of interest about shame in *medical* contexts. Shame permeates cultural media like medical memoir, medical reality television, and the trauma-directed narratives of medical television dramas. Often, this is for generic, situational reasons: the vulnerabilities and hierarchies of clinical encounters, sites of medical education, and healthcare cultures always contain a heightened risk of shame. These reasons are exploited because of an increased appetite for shame narrative, historical conditions related to the rise of memoir and confessional cultures, social media, and the belief that shame and shaming can work as regulating tools in situations of endemic inequality. Welcome developments like the scrutiny of medical authority, the increase in patient participation, and awareness of challenges like burnout bring with them the increased risk of shame: a risk that must not be avoided but engaged with, with care and sensitivity and compassion. We hope these essays will play a role in that continued conversation.

## Notes

1 Probyn, *Blush*, 130.

2 Probyn, *Blush*, 131.

3 Tyler, *Stigma*, 235; Foucault, *Birth of the Clinic*, 15–16.

4 Lazare, “Shame and Humiliation,” 1653; Davidoff, “Shame,” 623.

5 See Virant, “Chronic Illness and Shame”; Lazare, “Shame and Humiliation”; Bynum et al, “Shadow of Shame”; Dolezal, “Phenomenology of Shame”; Jaeb and Pecanac, “Shame in Patient-health Professional Encounters”; Woods, “On Shame and Voice-Hearing”; Inspire Charity, “If It’s Okay to Not Be Okay”; Miles, “Addressing Shame.”

6 See Dolezal and Lyons, “Health-Related Shame”; Dolezal and Gibson, “Beyond a Trauma-Informed Approach”; Dolezal and Bynum, “Shame Competence.”

7 Bewes, *Postcolonial Shame*, 15; Sheils and Walsh, “Introduction,” 1; Mitchell, *Writing Shame*, 24.

8 Sheils and Walsh, “Introduction,” 1.

9 Sheils and Walsh, “Introduction,” 1–2.

10 Mitchell, *Writing Shame*, 24.
